# Involvement of *MBD4* inactivation in mismatch repair-deficient tumorigenesis

**DOI:** 10.18632/oncotarget.5740

**Published:** 2015-10-16

**Authors:** Rossella Tricarico, Salvatore Cortellino, Antonio Riccio, Shantie Jagmohan-Changur, Heleen van der Klift, Juul Wijnen, David Turner, Andrea Ventura, Valentina Rovella, Antonio Percesepe, Emanuela Lucci-Cordisco, Paolo Radice, Lucio Bertario, Monica Pedroni, Maurizio Ponz de Leon, Pietro Mancuso, Karthik Devarajan, Kathy Q. Cai, Andres J.P. Klein-Szanto, Giovanni Neri, Pål Møller, Alessandra Viel, Maurizio Genuardi, Riccardo Fodde, Alfonso Bellacosa

**Affiliations:** ^1^ Cancer Epigenetics and Cancer Biology Programs, Fox Chase Cancer Center, Philadelphia, Pennsylvania, United States; ^2^ IFOM-FIRC Institute of Molecular Oncology, Milan, Italy; ^3^ Department of Cardiology, Boston Children's Hospital, Department of Neurobiology, Harvard Medical School, Boston, Massachusetts, United States; ^4^ Department of Pathology, Erasmus Medical Center Cancer Institute, Rotterdam, The Netherlands; ^5^ Department of Clinical Genetics and Department of Human Genetics, Leiden University Medical Center, Leiden, The Netherlands; ^6^ Department of Pathology and Laboratory Medicine, Medical University of South Carolina, Charleston, South Carolina, United States; ^7^ Cancer Biology and Genetics Program, Memorial Sloan Kettering Cancer Center, New York City, New York, United States; ^8^ Department of Systems Medicine, University of Rome Tor Vergata, Rome, Italy; ^9^ Medical Genetics Unit, Department of Mother & Child, University Hospital of Modena, Modena, Italy; ^10^ Institute of Medical Genetics, Catholic University of the Sacred Heart, “A. Gemelli” School of Medicine, Rome, Italy; ^11^ Department of Preventive and Predictive Medicine, Fondazione IRCCS Istituto Nazionale dei Tumori, Milano, Italy; ^12^ Department of Medicine and Public Health, University of Modena and Reggio Emilia, Modena, Italy; ^13^ Department of Medicine, Surgery and Neuroscience, University of Siena, Siena, Italy; ^14^ Department of Biostatistics, Fox Chase Cancer Center, Philadelphia, Pennsylvania, United States; ^15^ Department of Pathology, Fox Chase Cancer Center, Philadelphia, Pennsylvania, United States; ^16^ Inherited Cancer Research Group, Department for Medical Genetics, The Norwegian Radium Hospital, University Hospital, Oslo, Norway; ^17^ Experimental Oncology 1, CRO Aviano, National Cancer Institute, Aviano (PN), Italy

**Keywords:** MBD4/MED1, HNPCC, colorectal cancer, mismatch repair, mutations

## Abstract

The DNA glycosylase gene *MBD4* safeguards genomic stability at CpG sites and is frequently mutated at coding poly-A tracks in mismatch repair (MMR)-defective colorectal tumors (CRC). *Mbd4* biallelic inactivation in mice provided conflicting results as to its role in tumorigenesis. Thus, it is unclear whether *MBD4* alterations are only secondary to MMR defects without functional consequences or can contribute to the mutator phenotype. We investigated *MBD4* variants in a large series of hereditary/familial and sporadic CRC cases. Whereas *MBD4* frameshifts were only detected in tumors, missense variants were found in both normal and tumor DNA. In CRC with double-*MBD4*/MMR and single-*MBD4* variants, transition mutation frequency was increased, indicating that *MBD4* defects may affect the mutational landscape independently of MMR defect. *Mbd4*-deficient mice showed reduced survival when combined with *Mlh1^−/−^* genotype. Taken together, these data suggest that *MBD4* inactivation may contribute to tumorigenesis, acting as a modifier of MMR-deficient cancer phenotype.

## INTRODUCTION

MBD4, also known as MED1, is a methylcytosine binding domain (MBD)-containing, base excision repair (BER) thymine (T) and uracil (U) glycosylase that prevents mutability at CpG sites by removing T and U from G:T and G:U mismatches arising from spontaneous deamination of 5-methylcytosine (5mC) and cytosine (C), respectively [1–4]. Remarkably, MBD4 is also a binding partner of the mismatch repair (MMR) protein MLH1 and modulates the levels of core MMR proteins [2, 5]. In addition to its roles in genomic stability, MBD4 is a multifunctional protein involved in several cellular processes [6], including apoptotic response to DNA damage [5, 7], transcriptional repression [8], chromosomal stability [9] and Immunoglobulin Class Switch Recombination (CSR) [10]. More recently, a role of MBD4 in active DNA demethylation has been proposed but remains controversial [11–14].

Several studies focused on the role of alterations of the *MBD4* gene in tumorigenesis. *MBD4* is frequently mutated (20–45%) in hereditary and sporadic colorectal cancer (CRC) cases with MMR defects and consequent microsatellite instability (MSI) [15–18]. Moreover, a similar fraction of human endometrial, pancreatic and gastric carcinomas with microsatellite instability (MSI) also show *MBD4* mutations [17–21]. The majority of *MBD4* sequence variants found in these tumors are frameshift mutations that affect A_6_ and A_10_ polyadenine tracks in the coding region, leading to truncated MBD4 proteins that lack the glycosylase domain [15–19]. It has been proposed that these truncated MBD4 proteins lack the ability to bind MLH1 and may act in a dominant negative fashion, inhibiting the glycosylase activity of the wild type protein expressed from the unaffected allele [9, 22]. On the other hand, alternative mechanisms of biallelic *MBD4* inactivation have been described: in CRC frameshift mutations were accompanied by loss of heterozygosity (LOH) of the wild type allele [17]; and silencing of *MBD4* by promoter hypermethylation can occur in CRC and ovarian cancer [23]. An association between *MBD4* expression changes and DNA sequence variants has also been found in a fraction of hepatocellular carcinomas, brain tumors, esophageal squamous cell carcinomas and urothelial cell carcinomas [24–27].

Recently, in next-generation sequencing studies, *MBD4* somatic alterations, including point mutations and amplifications/deletions, have been identified, at a frequency ranging between 0.5%-8%, in a large series of unselected tumor samples (i.e. melanoma, ovarian, lung, esophagus and prostate cancers) [28–30]. However, it is still unclear whether *MBD4* alterations are due to the genomic instability, e.g. are secondary to a MMR defect without having functional consequences on the tumor mutational landscape, or can contribute to the mutator phenotype separately from the MMR defect, conferring a growth advantage to cancer cells.

Mouse studies have only partially clarified this issue. Biallelic inactivation of *Mbd4* alone does not initiate tumorigenesis in the mouse nor does it cause [31]. However, homozygous loss of *Mbd4* increases the frequency of C > T transition mutations at CpG sites and accelerates tumorigenesis in the Adenomatous Polyposis Coli (*Apc*) cancer-predisposing background [31, 32]. These results provide evidence that MBD4 functions *in vivo* to suppress mutations at CpG sites in mammalian genomes, and that its loss, while insufficient to initiate tumorigenesis by itself, can promote tumor formation in the context of a cancer-predisposing background. This contention was challenged by another study in which biallelic inactivation of *Mbd4* had no impact on mutation frequency and tumorigenesis in MMR-deficient tumors [33].

Thus, the role of *MBD4* in MMR-deficient tumorigenesis, which is precisely the situation in which most *MBD4* mutations in human cancer occur, is still a matter under debate. In order to elucidate the role of *MBD4* in colorectal tumorigenesis, we conducted a combined human-mouse study: we assessed the frequency, pattern and significance of germline and somatic *MBD4* mutations in a series of human CRC patients and tumors, respectively, and tested whether biallelic inactivation of *Mbd4* in a murine model may affect tumorigenesis and/or modify the tumor-predisposing phenotype on a cancer susceptible *Mlh1^−/−^* background.

## RESULTS

### Frequency and pattern of *MBD4* sequence variants

To investigate the role of *MBD4* in tumorigenesis and the interaction between *MBD4* and MMR genes, sequence analysis of the entire coding region of *MBD4* was performed in a total of 332 CRC cases, including 259 hereditary (i.e., meeting the Amsterdam/Bethesda criteria) or familial cases (partially fulfilling the Amsterdam/Bethesda criteria) and 73 sporadic cases. *MBD4* mutational analysis was initially conducted on 41 MMR-deficient MSI tumors, of which 17 were hereditary/familial and 24 were sporadic tumors. In terms of type of sequence change, frameshift mutations in coding A_6_ or A_10_ tracks were detected in hereditary/familial (8/17, 47.1%) and sporadic tumors (9/24, 37.5%), whereas one particular missense variant (p.Ala273Pro) was identified in 2/17 (11.8%) hereditary/familial and 1/24 (4.2%) sporadic tumors, respectively (Table 1).

**Table 1:** Frequency of somatic (tumor) *MBD4* variants according to clinical and molecular pathology data^1^

Type of *MBD4* sequence changes	Total number of tumors with *MBD4* variants (*n* = 20)	Frequency of *MBD4* variants in Hereditary/Familial CRC^2^ (*n* = 17)	Frequency of *MBD4* variants in Sporadic CRC (*n* = 24)
Missense	3	2/17	1/24
Frameshift mutation in coding A_6_ or A_10_ tracks	17	8/17	9/24

1 All the tumors included in this Table are MSI-H.

2 This group includes hereditary (fulfilling the Amsterdam or Bethesda criteria) and familial CRC cases (familial aggregation, partially fulfilling the Amsterdam or Bethesda criteria).

We next investigated *MBD4* sequence variants in the germline of hereditary/familial and sporadic CRC cases. While no germline frameshift changes were identified, *MBD4* missense variants were found in 11/242 (4.5%) hereditary/familial and 6/49 (12.2%) sporadic patients, respectively (Table 2). According to tumor MSI status, the patients on which germline sequence analysis was conducted were divided in three groups: i) MSI-H (*n* = 61); ii) MSS/MSI-L (*n* = 111); and iii) unknown MSI status (*n* = 119). Germline *MBD4* missense variants were present in MSI-H (8/61, 13.1%), MSS/MSI-L (4/111, 3.6%) and unknown MSI cases (5/119, 4.2%) (Table 2).

**Table 2:** Frequency of germline missense *MBD4* variants according to clinical and molecular pathology data

Total number of cases with *MBD4* variants	Frequency of *MBD4* variants in Hereditary/Familial CRC^1^ (*n* = 242)	Frequency of *MBD4* variants in Sporadic CRC (*n* = 49)	Frequency of *MBD4* variants in CRC with MSI-H (*n* = 61)	Frequency of *MBD4* variants in CRC with MSS/MSI-L (*n* = 111)	Frequency of *MBD4* variants in CRC with unknown MSI status (*n* = 119)
17	11/242	6/49	8/61	4/111	5/119

1 This group includes hereditary (fulfilling the Amsterdam or Bethesda criteria) and familial CRC cases (familial aggregation, partially fulfilling the Amsterdam or Bethesda criteria).

**Figure 1:**
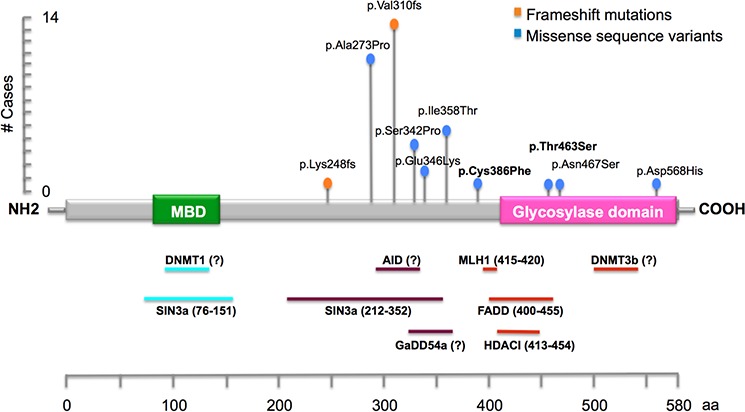
Schematic illustration of *MBD4* gene, showing the main known functional and putative domains, and location of the identified DNA variants in this study The novel variants identified in this study are reported in bold. The recurrent frameshift mutations (p.Lys248fs and p.Val310fs) are located in coding A_6_ and A_10_ tracks.

Overall, we found a total of 8 germline/somatic *MBD4* missense variants in 20 patients/tumors, of which 2 missense variants (p.Cys386Phe, p.Thr463Ser) were identified for the first time in this study (Fig. 1) (Table 3). Co-occurrence of *MBD4* variants (frameshift or missense changes) with MMR mutations was found in 17/332 (5.1%) total patients/tumors, and in 28/102 (27.4%) of MSI-H patients (Table 2)/tumors (Table 1). Germline and somatic p.Ala273Pro variant was found associated with MMR defects in 3 patients and 2 tumors, respectively; of these five cases, two were associated with *MLH1* pathogenic mutations, two with *MSH2* pathogenic mutations and one with a *MSH6* variant of unknown significance (Table 3). Co-occurrence of *MLH1* pathogenic mutations (8 germline and 1 somatic) and the recurrent *MBD4* frameshift mutations in coding A_6_ or A_10_ tracks were identified in 9 tumors. The *MBD4* variants p.Asp568His and p.Cys386Phe were found in *MLH1*-deficient tumors, and the *MBD4* p.Ser342Pro variant was found in a *MSH2*-deficient tumor. No co-occurrence with MMR defects was observed for the remaining *MBD4* variants (p.Glu346Lys, p.Ile358Thr, p.Thr463Ser, p.Asn467Ser) (Table 3).

**Table 3:** Classification and relationship to MMR status of *MBD4* variants

Exon	*MBD4* sequence change^1^	Type and number of sequence changes	Frequency in control chromosomes (%)^2,3^	Functional or Interaction Domains	Glycosylase Assay^3^	*In Silico* Predictions^4^	Classification^5^	Co-occurrence of MMR germline defect
3	c.811G > A (p.Ala273Pro) *rs10342*	5 germline +3 somatic^6^	8.11 8.11	SIN3a/HDAC1	ND	4/4 Concordant B	likely non-pathogenic	*MLH1* (*n* = 2) *MSH2* (*n* = 2) *MSH6* (*n* = 1)
	c.1024T > C (p.Ser342Pro) *rs2307289*	2 germline	4.31 6	SIN3a/HDAC1	slightly reduced	4/4 Concordant B	VUS	*MSH2* (*n* = 1)
	c.1036G > A (p.Glu346Lys) *rs140693*	2 germline	0.99 11	SIN3a/HDAC1	ND	3/4 Concordant B	likely non-pathogenic	none
	c.1073T > C (p.Ile358Thr) *rs2307298*	4 germline	0.86 0.4	linker region	ND	4/4 Concordant B	likely non-pathogenic	none
	**c.1158G > T** **(p.Cys386Phe)**	1 germline	NR	linker region	slightly reduced	3/4 Concordant P	VUS	*MLH1*
5	**c.1387 > T** **(p.Thr463Ser)**	1 germline	NR	glycosylase domain	markedly reduced	3/4 Concordant P	likely pathogenic	none
	c.1400 > G (p.Asn467Ser) *rs78782061*	1 germline	0.17 0.17	glycosylase domain	ND	Discordant	VUS	none
8	c.1702G > C (p.Asp568His) *rs2307293*	1 germline	0.44 0.44	glycosylase domain	proficient	Discordant	VUS	*MLH1*

1 Novel variants identified in this study are reported in bold

2 Minor allele frequencies (MAF) obtained using Exome Variant Server (top number) and 1000 Genomes browser (bottom number)

3 NR = not reported; ND = not done

4 P = pathogenic; B = benign

5 VUS = Variant of Unknown Significance

6 Of these, one tumor carries a germline *MLH1* mutation, one tumor carries a germline *MSH2* mutation and one tumor carries a germline *MSH6* variant of unknown significance.

For twenty cases, matched normal and tumor DNA was available, allowing us to conduct LOH studies with markers *D3S3606, D3S1587* and *D3S1290* at the *MBD4* locus (3q21-22), as previously described [17]. Out of six tumors found to exhibit LOH at 3q21–22, three tumors displayed a frameshift mutation at the A_10_ track (Suppl. Fig. 1).

### Functional and *in silico* assays of *MBD4* missense variants

**Figure 2:**
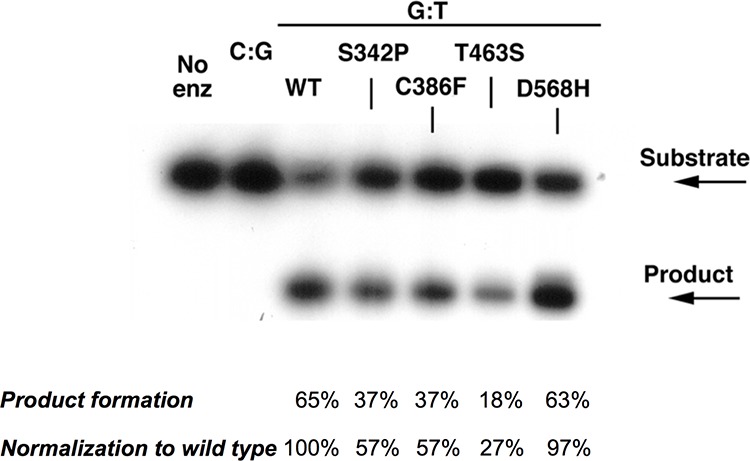
Single-turnover thymine glycosylase assays for MBD4 DNA coding variants Wild-type, p.Ser342Pro, p.Cys386Phe, p.Thr463Ser and p.Asp568His recombinant MBD4 proteins were assayed for glycosylase activity. The p.Thr463Ser mutant protein showed a marked reduction of thymine glycosylase activity for G:T mismatch (lane 6).

To gain insight into the functional consequences of the *MBD4* missense variant p.Thr463Ser and p.Asp568His located in the glycosylase domain, we carried out *in vitro* glycosylase assays to assess the rate of thymine removal from double-stranded DNA containing a G:T mismatch. Wild type MBD4, p.Thr463Ser and p.Asp568His mutant proteins, as well as two additional missense variants (p.Ser342Pro and p.Cys386Phe) not located in the glycosylase domain were tested. Compared with the wild type protein, only the mutant protein p.Thr463Ser showed a significant reduction in the rate of thymine removal from the G:T substrate (approximately 18% of wild type protein). Moreover, the p.Ser342Pro and p.Cys386Phe mutant proteins showed slightly reduced glycosylase activity (approximately 37% of wild type protein) (Fig. 2).

*In silico* analysis was also performed to predict the pathogenic role of the coding variants identified in this study. Three out of four programs predicted a possible impact of p.Cys386Phe and p.Thr463Ser amino acid substitutions on MBD4 function and a non-pathogenic role for p.Glu346Lys. Conversely, they concordantly predicted a non-pathogenic role for p.Ala273Pro, p.Ser342Pro and p.Ile358Thr variants. Discordant predictions were obtained for the two remaining variants, p.Asn467Ser and p.Asp568His. In addition, p.Ala273Pro, p.Ser342Pro, p.Glu346Lys, p.Ile358Thr, p.Asn467Ser and p.Asp568His were found in control populations (Table 3).

Although the *in silico* analysis predicted that p.Ala273Pro, p.Ser342Pro and p.Glu346Lys would not affect MBD4 protein function, it is possible that they could alter the interaction of MBD4 with Sin3A and HDAC1 proteins, which bind within this region [8] (Table 3).

In order to test the hypothesis that *MBD4* alterations are not only secondary to MSI in MMR-deficient tumors and may be selected for during tumorigenesis, we evaluated the frequency of pathogenic and likely non-pathogenic *MBD4* variants, as determined by *in silico* prediction, in a cohort of unselected CRC cases (*n* = 1536), from COSMIC and cBioPortal databases, and control samples (*n* = 6503), from the Exome Variant Server (http://evs.gs.washington.edu/EVS/). Pathogenic *MBD4* variants were found in 13/1536 (0.85%) and 10/6503 (0.15%) CRC cases and controls, respectively; likely non-pathogenic variants were present in 21/1536 (1.37%) and 24/6503 (0.37%) CRC cases and controls, respectively. A test of association between pathogenic *MBD4* variants and presence of tumor was highly significant (Fisher's exact test *p*-value <0.0001). Moreover, a binomial test of proportions between the frequency of *MBD4* pathogenic and likely non-pathogenic variants in tumor and control samples was also significant (*p*-value < 0.0001).

### Higher frequency of transition mutations in CRC tumors with *MBD4* variants

**Figure 3:**
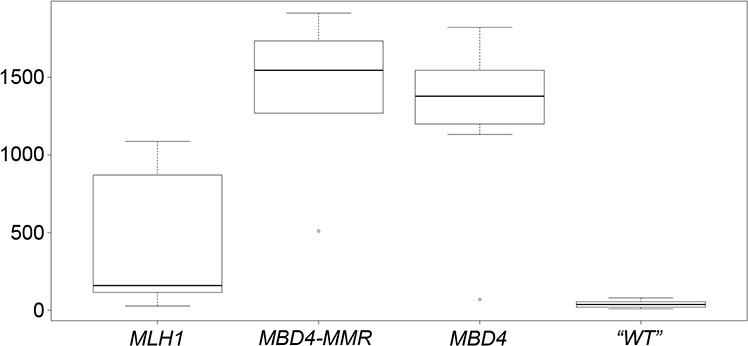
Box-and-whisker plot of the number of C:G > T:A transition mutations in *MLH1, MBD4-MMR, MBD4* and “WT” CRC groups In each plot, the height of the box represents the inter-quartile range (IQR) where the upper and lower ends indicate the third and first quartiles, respectively. The solid black horizontal line inside the box represents the median value while the whiskers (the two solid horizontal lines at either end, connected by dotted lines) extend to the most extreme data points which are no more than 1.5 times the IQR from the box in each direction (the points that lie beyond these whiskers are considered to be outliers). Statistical significance of the comparisons is as follows: *MLH1* vs *MBD4*: *p* = 0.0064; *MLH1* vs *MBD4-MMR*: *p* = 0.0054; *MLH1* vs *“WT”*: *p* = 0.2019; *MBD4* vs *MBD4-MMR*: *p* = 0.67; *MBD4* vs *“WT”*: *p* = 1.03 × 10^−6^; *MBD4-MMR* vs *“WT”*: *p* < 1 × 10^−6^.

Because an *MBD4* defect is expected to lead to C:G > T:A transition mutations in the context of CpG sites [3], we analyzed the data deposited in the COSMIC database v72 [34] to investigate the average number of C:G > T:A transitions in CRC tumors with *MBD4* pathogenic (frameshift) variants, *MLH1* pathogenic variants or combined *MBD4* plus major MMR gene variants (*MLH1, MSH2* and *MSH6*) and, as controls, CRC tumors with no *MBD4* or major MMR gene variants. We found that the average number of C:G > T:A transitions in CRC with combined *MBD4* plus major MMR gene variants, CRC with *MBD4* variants, and CRC with *MLH1* variants was 35, 31.4 and 10.6 times higher than control tumors, respectively (Fig. 3). Although this analysis is not limited to CpG sites and may reflect an overall increase in the transition load, these observations suggest that *MBD4* variants may modify the pattern of somatic mutations in CRC, i.e. act as transition mutators, separately from MMR defects.

### Deficiency of *Mbd4* leads to accelerated tumorigenesis in *Mlh1^−/−^* mice

**Figure 4:**
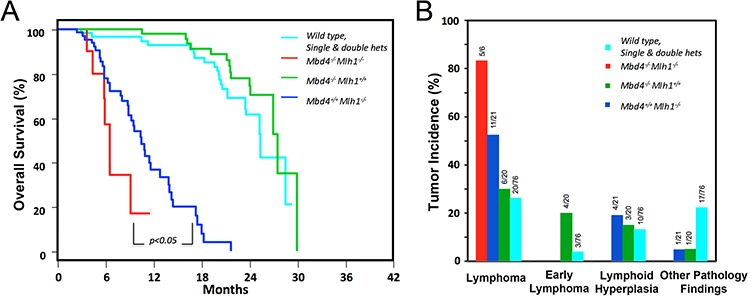
*Mbd4* deficiency alters tumorigenesis in *Mlh1^−/−^* mouse model **A.** Kaplan-Meier analysis of *Mbd4^−/−^*Mlh1*^−/−^* mice (*n* = 10), *Mbd4^−/−^*Mlh1*^+/+^* mice (*n* = 22) and *Mbd4^+/+^*Mlh1*^−/−^* (*n* = 24) and wild-type, single and double heterozygous mice (*n* = 122) revealed a significant reduction of survival in *Mbd4^−/−^*Mlh1*^−/−^* mice (*p* value < 0.05) for the comparison of *Mbd4^−/−^*Mlh1*^−/−^* mice vs. *Mbd4^+/+^*Mlh1*^−/−^* mice). **B.** *Mbd4* biallelic inactivation increased lymphoma incidence in *Mbd4^−/−^*Mlh1*^−/−^* double knockout mice when compared with *Mlh1^−/−^* single knockout mice, but this difference was not statistically significant by Fisher's exact test (*p* = 0.35).

In order to better characterize the *in vivo* role of biallelic inactivation of *Mbd4* in the context of MMR-deficient tumorigenesis, we generated *Mbd4- Mlh1-* double knock-out mice. A total of 178 mice were divided into four cohorts: *Mbd4*^−/−^ *Mlhl*^−/−^, *Mbd4*^+/+^ *Mlhl*^−/−^, *Mbd4*^−/−^ *Mlhl*^+/+^ and a cohort of wild type, single or double heterozygotes together. Mice were aged and monitored for lymphoma development, which is the predominant tumor type in this *Mlh1*-mutant strain [35]. *Mbd4* deficiency conferred a significant survival reduction in *Mbd4^−/−^*Mlh1*^−/−^* double knock-out mice compared with *Mlh1^−/−^* single knock-out mice (*p*-value < 0.05) (Fig. 4a). No significant differences in survival were observed between the cohorts of *Mbd4^−/−^*Mlh1*^+/+^* and wild type, single and double heterozygous mice (Fig. 4a).

**Figure 5:**
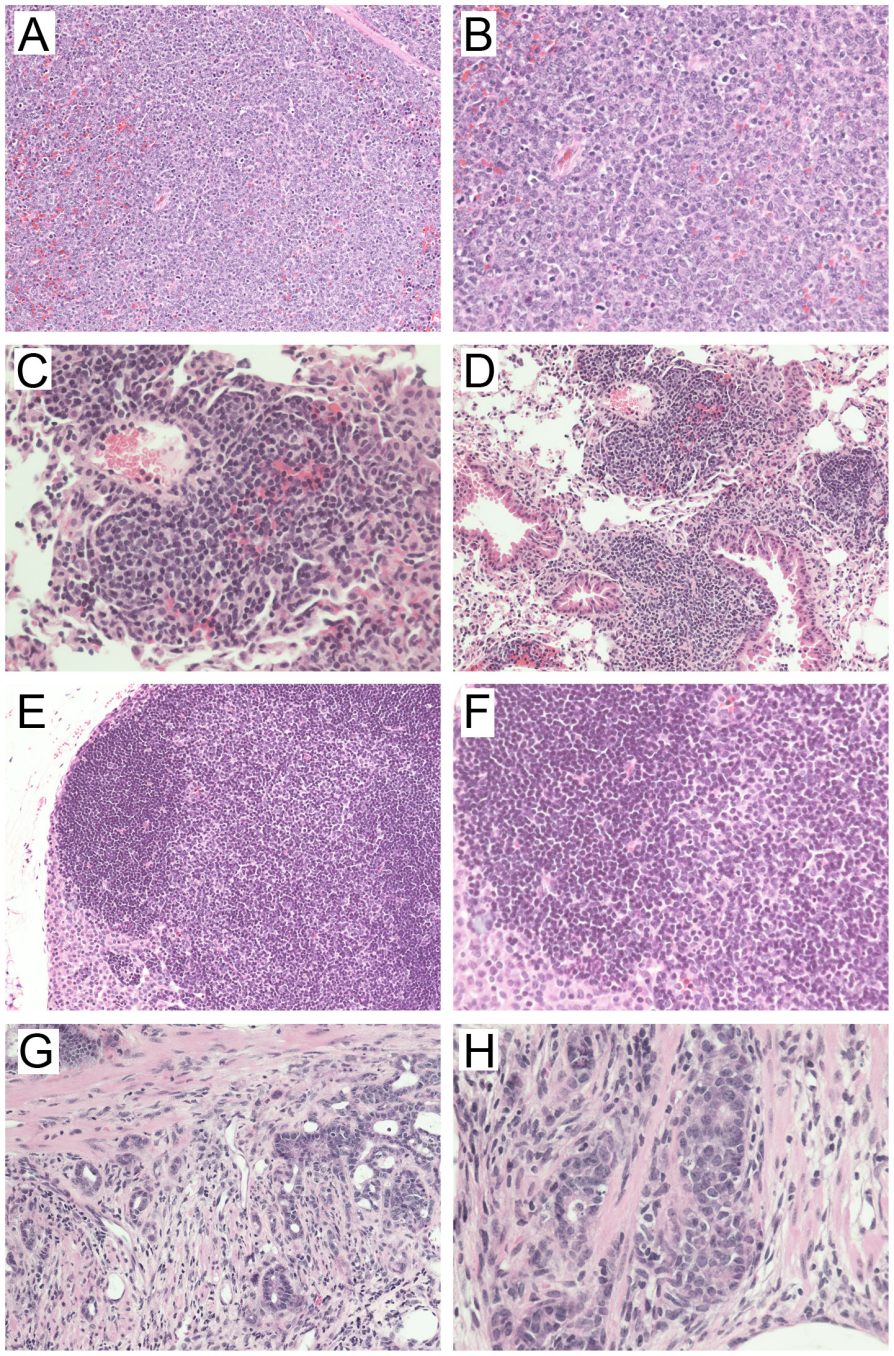
Representative hematoxylin & eosin-stained sections of tumors developed in A-B) *Mbd4^−/−^*Mlh1*^−/−^*; C-D) *Mbd4^−/−^*Mlh1*^+/+^* ; E-F) *Mbd4^+/+^*Mlh1*^−/−^*; and G-H) *Mbd4^+/−^*Mlh1*^−/−^* mice Panels **A)** and **B)** show a high-grade lymphoma typical of the double mutant mice. Panels **C)** and **D)** depict severe lymphoid hyperplasia in the lung characterized by an abundant perivascular lymphocytic infiltrate. Panels **E)** and **F)** show lymphoid hyperplasia in a lymph node. Panels **G)** and **H)** show fields of invasive adenocarcinoma of the intestine (note foci of invasion in the muscularis in panel **H)**). 20X magnification (left panels) and 40X magnification (right panels) are shown.

In keeping with the survival differential, a difference in tumor incidence and distribution was observed in *Mbd4*^−/−^ *Mlh1*^−/−^ double knock-out mice compared with *Mlh1* single knockout mice (Fig. 4b). Specifically, *Mbd4*^−/−^ *Mlh1*^−/−^ mice developed lymphomas at a higher incidence (83%) than *Mlh1^−/−^* mice (52%), but this difference was not statistically significant (Fisher's exact test *p* = 0.35). A detailed histological analysis revealed that tumor spectrum associated with the *Mlh1* defect was modified in double-mutant mice, which developed only high-grade lymphomas compared to *Mlh1* single knockout mice, which most frequently manifested high grade lymphoma (52%), but also lymphoid hyperplasia (19%) and other tumors (5%) (Fig. 5a–5d).

No significant differences in tumor incidence and distribution were found between *Mbd4^−/−^*Mlh1*^+/+^* mice and the other genotype groups, monitored over a period of 30 months. Specifically, lymphomas were identified in 30% of *Mbd4^−/−^*Mlh1*^+/+^* mice, a frequency marginally higher than that of mice with the other genotypes (26%). Early lymphomas were found in 20% of *Mbd4^−/−^*Mlh1*^+/+^* and 4% of *Mbd4* wild type, single and double heterozygous mice; lymphoid hyperplasia was found in 15% and 13% of *Mbd4^−/−^*Mlh1*^+/+^* (Fig. 5e–5f) and the remaining cohort, respectively. Finally, additional pathology findings, including intestinal adenocarcinoma (Fig. 5g–5h), were identified in 5% and 22% of *Mbd4* single knockout mice and the remaining genotypes, respectively.

## DISCUSSION

In this study, we first analyzed the frequency, pattern and significance of germline/somatic *MBD4* sequence variants in a large series of human hereditary/familial and sporadic CRC patients/tumors, and found that *MBD4* frameshift mutations are only detected in tumors (somatic change), whereas missense variants are detected, at lower frequency, in both normal and tumor DNA. Evaluation of different molecular, *in silico* and functional parameters (including: impaired glycosylase activity, concordance of at least three out of four software predictions, and frequency in control chromosomes), provided evidence on the biological role of 4 out of 8 *MBD4* missense variants identified in this study. Specifically, one variant (p.Thr463Ser) is considered as likely pathogenic and the other three variants (p.Ala273Pro, p.Glu346Lys and p.Ile358Thr) as likely non-pathogenic. The remaining 4 missense variants are considered as variants of unknown significance (VUS), due to the lack of data available for the classification or discrepancies among the parameters evaluated here (Table 3).

Based on the glycosylase assay, the p.Thr463Ser variant has significantly decreased activity. The p.Ser342Pro and p.Cys386Phe variants have slightly reduced activity despite not mapping in the glycosylase domain; we cannot rule out a possible impact of altered protein folding on the pathogenic role of these two variants. Recently, biochemical studies have shown that MBD4 p.Asp568His mutant protein has reduced catalytic activity and binding affinity to DNA [36]. This variant showed apparently minimal impact on glycosylase activity in our biochemical assay and scored as non-pathogenic in two out of four prediction programs (Mutation Assessor and SIFT). The reasons for this discrepancy are presently unknown.

Our findings indicate that *MBD4* inactivation may occur not only by expansions/deletions in the polyadenine tracks in MSI-H tumors, as previously reported [15–18], but also by point mutations in other portions of the coding sequence. The occurrence of *MBD4* missense changes and the higher frequency of *MBD4* pathogenic variants in CRC genomic data, compared to controls, suggests that *MBD4* mutations are not only secondary to MSI in MMR-deficient tumors, but may occur independently and presumably be selected for during tumorigenesis. Moreover, we have found that the overall frequency of *MBD4* mutations in MSI-H tumors (28/102, 27.4%) is in the range of the mutation frequencies (19.4–92%) of the genes contributing to the mutator phenotype in CRC tumorigenesis [37]. Similarly, a maximum likelihood method to identify real target genes of MMR also indicated that *MBD4* frameshift mutations may provide selective pressure during CRC tumorigenesis [38]. In a portion of our tumors, we found evidence of *MBD4* frameshift mutations and LOH at its locus on 3q21-q22, suggesting biallelic inactivation (Suppl. Fig. 1), as previously proposed [17]. All these data support the notion that *MBD4* defects may further increase genomic instability and play a role in colorectal tumorigenesis.

The possibility that *MBD4* may act as a mutator in intestinal tumorigenesis is confirmed by two studies showing that *Mbd4* inactivation in reporter BigBlue mice leads to a 3-fold increase in mutation frequency, especially C > T transition at CpG sites, in the spleen and small intestine [31, 32]. This enhanced mutability manifests also as accelerated tumorigenesis due to *Mbd4* loss in two *Apc* mutant mouse models [31, 32]. A pathogenic role of *MBD4* variants and their effect on the tumor mutational landscape, possibly consistent with its role in avoiding mutability at CpG sites [3, 32, 33], is confirmed by our finding of increased frequency of C:G > T:A transitions in CRC cases with *MBD4* mutation or combined *MBD4* plus major MMR gene mutations, compared to both CRC cases with MLH1 variants and CRC cases with no mutation in MBD4 or major MMR gene (Fig. 3). Future studies will have to determine the sequence context of C:G > T:A transitions in CRC cases with *MBD4* mutation, in order to ascertain whether these transitions occur preferentially in the context of CpG sites, as predicted on the basis of MBD4 anti-mutagenic function. Importantly, C:G > T:A transition mutations in the context of CpG sites are the most prevalent mutational signature in the vast majority of human cancer types, including CRC [39].

Here, we evaluated the contribution of *Mbd4* inactivation *on an Mlh1*-cancer predisposing background *in vivo*. We found that *Mbd4* and *Mlh1* double knockout mice showed a significant survival reduction and increased incidence of high-grade lymphomas compared with *Mlh1* single knockout mice. Noteworthy, our findings differ from a previous study, in which biallelic inactivation of *Mbd4* had no impact on mutation frequency *in vivo* and did not modify the cancer predisposition phenotype in mice doubly deficient for *Mbd4* and MMR genes [33]. This discrepancy could be due to the fact that the targeted allele used in the previous study may allow the synthesis of a small amount of wild type mRNA [32]. Additionally, our data are based on an accurate study of a large number of mice (*n* = 178) compared to the smaller number (*n* = 57) included in the previous study [33]. The relatively high incidence of lymphoma, early lymphoma and lymphoid hyperplasia in *Mbd4*^−/−^ mice may be due to the role of this gene in Immunoglobulin CSR [10]. Consistent with a possible pathogenic role, our detailed histological analysis revealed that *Mbd4* modifies the tumor spectrum associated with the *Mlh1* defect leading to more aggressive tumors. Thus, loss of *Mbd4* function confers increased tumor susceptibility and a more severe outcome when combined with the cancer-predisposing *Mlh1^−/−^* background.

Moreover, our results that *MBD4* alterations may contribute to MMR-deficient tumorigenesis are supported by our previous study showing reduction in MMR protein levels in *Mbd4^−/−^* MEFs [5]. More recently, additional evidences have supported the role of *MBD4* in CRC tumorigenesis. In fact, overexpression of a truncated form of MBD4 lacking the glycosylase domain in a BigBlue-transfected human *MSH6*-deficient colorectal cancer cell line, led to a 2-fold increase in mutation frequency and predisposed to chromosomal instability, compared to controls [9, 22].

Taken together, our evidence indicates a key role for *MBD4* as a modifier of tumorigenesis associated with MMR mutations, likely by increasing the genomic instability phenotype of a subset of MMR-defective tumors, specifically contributing to elevated C:G > T:A transitions. Our results also suggest that *MBD4* mutations may be responsible for a worse outcome (based on the mouse studies), and possibly resistance to therapy [5, 7], of a subset of MMR-deficient tumors. A retrospective analysis in a larger series of human samples with MMR and *MBD4* defects may be helpful to confirm the association with the more severe outcome observed in our *in vivo* study.

## MATERIAL AND METHODS

### Patient selection and *MBD4* molecular analysis

This study was performed on sporadic and hereditary/familial CRC cases from high-risk patients referred for genetic counseling to the following institutions: Departments of Medical Genetics and Pathology of the University of Helsinki, Centro di Riferimento Oncologico of Aviano, Istituto Nazionale Tumori (INT) of Milan, Department of Human Genetics of the Leiden University Medical Center (LUMC), Josephine Nefkens Institute of Erasmus MC of Rotterdam and Department of Medical Genetics of the Catholic University of Rome. The presence of MMR defects was ascertained by a combination of MSI testing, immunohistochemistry for MLH1 and MSH2, sequencing and Multiplex ligation-dependent probe amplification (MLPA) of *MLH1* and *MSH2*, as previously done [17, 40–42]. LOH at the *MBD4* locus (3q21-22) in tumor DNA was performed as previously described [17]. Clinical and histopathogical data were also collected during genetic counseling.

A total of 332 hereditary (defined as meeting the Amsterdam/Bethesda criteria), familial (partially fulfilling the Amsterdam/Bethesda criteria) and sporadic CRC cases were selected for *MBD4* sequence analysis. Tumors were classified as microsatellite stable (MSS) or with MSI-high (MSI-H) or -low (MSI-L), according to standard methods [43–45]. In this study, we also included 42 tumors previously reported [17], re-evaluated for MSI status and/or MMR defects. The complete coding sequence and flanking exon-intron borders of the *MBD4* gene were investigated by direct sequencing of PCR products from genomic DNA extracted from peripheral blood and fresh or paraffin-embedded tumor tissues. Primers and PCR conditions are available upon request.

*MBD4* variants were defined according to the recommendations of the Human Genome Variation Society (http://www.hgvs.org/mutnomen/). DNA mutation numbering is based on the *MBD4* cDNA sequence (GenBank accession numbers NM_003925.2) with the A of the ATG translation-initiation codon numbered as +1. Amino acid numbering starts with the translation initiator methionine as +1.

The study was performed in accordance with the Declaration of Helsinki (http://www.wma.net/en/30publications/10policies/b3/index.html). Informed consent was obtained from all patients for the use of specimens and clinico-pathological data for research purposes, according to the guidelines established by the local ethical committee.

The Exome Variant Server (http://evs.gs.washington.edu/EVS/) and 1000 Genomes databases (www.1000genomes.org/) were used to ascertain the frequencies of the *MBD4* variants in control populations.

### Functional and *in silico* assays

DNA *N*-glycosylase assays were performed for the following DNA coding variants: p.Ser342Pro, p.Cys386Phe, p.Thr463Se and p.Asp568His. Site-directed mutagenesis of *MBD4* cDNA for the above-mentioned DNA variants was carried out using the QuickChange mutagenesis kit (Stratagene). Cloning, expression and purification of wild type and mutant MBD4 proteins were performed as previously described [46, 47]. To determine the effect of the MBD4 mutant proteins on the intrinsic rate of thymine and uracil removal compared with wild-type enzyme, glycosylase assays were carried out under single-turnover conditions, using a 37-bp duplex containing a centrally located G:T mismatch in a CpG context [46, 47].

Putative effects of *MBD4* missense variants were evaluated at the protein level using PolyPhen-2 (http://genetics.bwh.harvard.edu/pph2/) [48], SIFT (http://sift.jcvi.org/) [49], Mutation Taster (http://www.mutationtaster.org/) [50] and Mutation Assessor (http://mutationassessor.org) [51].

The frequency of C:G > T:A transition mutations was evaluated in four series of CRC samples selected from the COSMIC dataset v72 [34]: i) tumors with *MBD4* pathogenic (splicing, truncating and nonsense) variants (*n* = 7); ii) tumors with *MBD4* variants plus variants in one of the major MMR genes (i.e. *MLH1, MSH2* and *MSH6*) (*n* = 5); iii) tumors with pathogenic (splicing, truncating and nonsense) *MLH1* variants (*n* = 11); tumors with no *MBD4* or MMR variants (*n* = 12).

### Generation of *Mbd4^−/−^*Mlh1*^−/−^* double mutant mice

The *Mbd4* Δ2–5 mutant mice used in this study were generated by targeted deletion of exons 2–5 and are null for both 5mC binding and glycosylase activity [5]. *Mbd4*^+/−^ mice were mated with *Mlh1*^+/−^ mice [35] to generate F_1_ double heterozygotes (*Mbd4*^+/−^ *Mlh1*^+/−^); both strains have a C57BL/6 genetic background. F_1_ double heterozygotes were interbred to generate F_2_ mice with experimental and control genotypes: *Mbd4*^−/−^ *Mlhl*^−/−^, *Mbd4*^+/+^ *Mlhl*^−/−^, *Mbd4*^−/−^ *Mlhl*^+/+^ and *Mbd4*^+/+^ *Mlhl*^+/+^ mice. Animal protocols and all the procedures of mouse handling were approved by the Institutional Animal Care and Use Committee of the Fox Chase Cancer Center.

### Histopathological and tumor incidence analyses

Animals were sacrificed at the first development of signs of distress or when tumor growth became apparent. Tumors and other organs (including spleen, lymph nodes, GI tract and liver) were embedded in paraffin, and sections were stained with hematoxylin/eosin. A total of 178 mice, divided in four cohorts based on genotypes (*Mbd4*^−/−^ *Mlhl*^−/−^, *Mbd4*^+/+^ *Mlhl*^−/−^, *Mbd4*^−/−^ *Mlhl*^+/+^ and a cohort of wild type, single or double heterozygotes together), were included in this study and the incidence and the spectrum of tumors were evaluated. A Kaplan-Meier analysis was performed to plot the overall survival of the mice. Statistical significance was measured using the log-rank test.

### Statistical methods

COSMIC dataset v72 [28], cBioPortal [29] and Exome Variant Server (http://evs.gs.washington.edu/EVS/) data were also used to ascertain the frequency of *MBD4* pathogenic and likely non-pathogenic variants in colorectal cancer tumors and controls. Fisher's exact test and a binomial test of proportions were used to compare the frequency of *MBD4* pathogenic and likely non-pathogenic changes in tumor and control samples. Both tests were two-sided with a Type I Error of 0.05 to determine statistical significance.

Fig. 3 displays the distribution of the number of C > T substitutions in four series of CRC samples (see above): *MLH1, MBD4-MMR, MBD4* and “wild type (WT)” control (no *MBD4* or MMR gene mutations). In order to account for over-dispersion in counts, a quasi-Poisson model was used to compare the mean number of C > T substitutions between *MBD4-MMR* and *MBD4, MBD4-MMR* and WT, *MBD4-MMR* and *MLH1, MLH1* and *MBD4,* and between *MLH1* and *WT*. Fisher's exact test was used to compare the incidence of lymphomas between *Mbd4*^−/−^ *Mlh1*^−/−^ and *Mbd4*^+/+^ *Mlh1*^−/−^ mice. All tests were two-sided and a Type I Error of 0.05 was used to determine statistical significance.

## SUPPLEMENTARY FIGURE


