# Supplementary material for: Involvement of MBD4 inactivation in mismatch repair-deficient tumorigenesis
Source: Oncotarget. 2015 Oct 16;6(40):42892–904. doi: 10.18632/oncotarget.5740 (PMC4767479; doi:10.18632/oncotarget.5740)
Supplement: Supplementary file 1 [file oncotarget-06-42892-s001.pdf]

SUPPLEMENTARY FIGURE

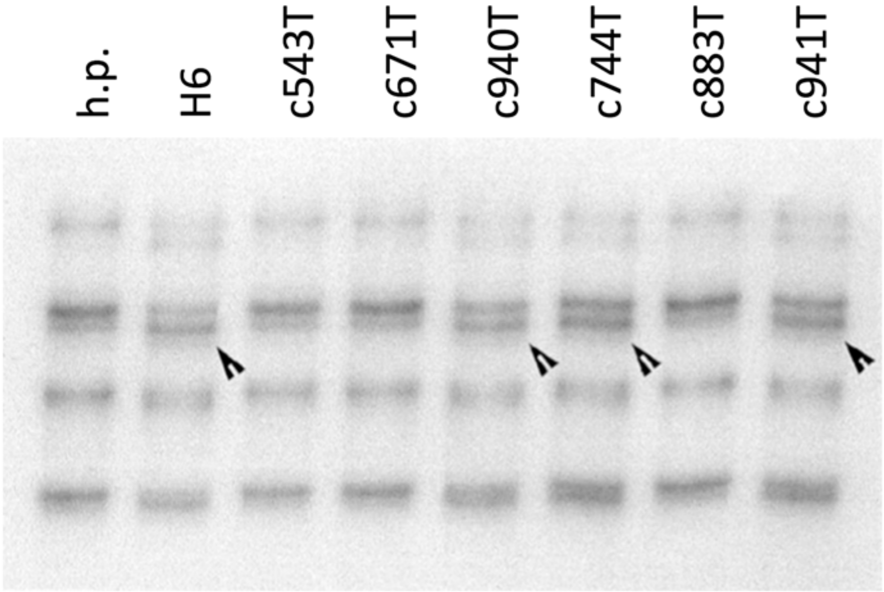

Supplementary Figure S1: Mutations of *MBD4* at coding $(A)_{10}$ track in six CRC cases exhibiting loss of heterozygosity at the *MBD4* locus (3q21–22). Single Strand Conformational Polymorphism (SSCP) analysis is shown for CRC samples, HCT116 cell line (H6, positive control) and normal human placental DNA (h.p., negative control). Arrowheads indicate prominent abnormally migrating bands.
